# Cytoreductive Nephrectomy Following Immunotherapy: Evolution, Pearls, and Pitfalls of Treatment

**DOI:** 10.33696/immunology.6.202

**Published:** 2024

**Authors:** Laura E. Davis, Adam Calaway, Eric A. Singer, Shawn Dason

**Affiliations:** 1 Case Western Reserve University Hospitals Urology Institute, Cleveland, OH, USA; 2 Division of Urologic Oncology, The Ohio State University Comprehensive Cancer Center, Columbus, OH, USA

**Keywords:** Cytoreductive nephrectomy, Metastatic renal cell carcinoma, Perioperative outcomes, Immune checkpoint inhibition, Tyrosine kinase inhibition

## Abstract

**Introduction:**

Renal Cell Carcinoma (RCC) is among the most frequently diagnosed malignancies in both genders with over 81,000 estimated cases in 2024. Despite increasing incidence of renal cell carcinomas <4 cm, up to 1/3 of patients diagnosed with RCC exhibit metastatic disease (mRCC) at time of diagnosis. Cytoreductive nephrectomy (CN), a procedure which encompasses the surgical removal of the primary tumor in patients with metastatic disease, was offered upfront as standard of care during the cytokine era; however, as systemic treatment has evolved, the role of CN in mRCC patients has become less clear.

**Purpose of Review:**

We sought to review the evolution of CN in mRCC patients from historical treatments through current standard of care considering ongoing clinical trials and perioperative considerations for CN in patients treated with tyrosine kinase inhibitors (TKI) and immune checkpoint inhibitors (ICI).

**Conclusion:**

CN following immunotherapy is safe and beneficial in appropriately selected patients. The choice to perform CN in patients with mRCC amidst an ever-changing treatment landscape is nuanced. Clinical trial enrollment is critical to refine selection criteria and timing of CN. As treatment options continue to progress, shared decision-making and multidisciplinary collaboration remain paramount in selecting the optimal treatment course for each patient.

## Introduction

Renal Cell Carcinoma (RCC) is among the most frequently diagnosed malignancies in both genders with over 81,000 estimated cases in 2024 [1]. Despite the increasing incidence of small renal cell carcinomas, up to 1/3 of patients diagnosed with RCC exhibit metastatic disease (mRCC) at time of diagnosis [2]. In spite of a rapid evolution of mRCC treatments over the last two decades, consensus on optimal multimodal treatment, particularly regarding the role of cytoreductive nephrectomy (CN) in this patient population is unclear. We seek to review the evolution of CN in mRCC patients from historical treatments through current standard of care giving particular consideration to ongoing clinical trials and perioperative considerations.

## Evolution of Systemic Therapies

Prior to the mid 2000s, cytokine therapy with interleukin-2 (IL-2) or interferon alpha-2b (INF) was the mainstay treatment for mRCC. However, these immunomodulatory drugs exhibited a response rate less than 15% [3]. Cytoreductive nephrectomy (CN), a procedure which encompasses the surgical removal of the primary tumor in patients with metastatic disease, was offered upfront as standard of care during the cytokine era.

Results of two randomized controlled trials (RCT) which reported in the early 2000s supported this algorithm. One of these, SWOG 8409, compared patients with mRCC randomized to CN followed by INF vs INF alone with OS as primary endpoint. Patients treated with surgery and INF had superior outcomes compared to those treated with INF alone (mOS: 11 vs 8 months, p=0.05) [7]. The beneficial effects of CN were confirmed in a trial by EORTC which randomized patients with mRCC to surgery followed by INF vs INF alone. Results favored the CN group for both outcomes (time to progression: 5 vs 3 months, HR 0.60, 95% CI 0.36–0.97; mOS: 17 vs 7 months, HR 0.54, 95% CI 0.31–0.94) [5].

Beginning with the approval of sorafenib and sunitinib in 2006, systemic therapy for mRCC shifted towards tyrosine kinase inhibitors (TKIs) which proved to be more effective than cytokine therapy. The role of CN in combination with TKIs was investigated in two RCTs—CARMENA and SURTIME.

CARMENA, published in 2018, was the first trial to compare the TKI sunitinib alone to CN and sunitinib for patients with Memorial Sloan Kettering Cancer Center (MSKCC) intermediate or poor risk mRCC with a primary endpoint of OS. Results revealed non-inferiority for treatment with sunitinib alone (stratified HR, 0.89; 95% CI, 0.71 to 1.10; upper boundary of the 95% CI for noninferiority, ≤1.20). The sunitinib group exhibited a longer mOS of 18.4 months (95% CI, 14.7 to 23.0) vs 13.9 months in the CN + sunitinib group (95% CI, 11.8 to 18.3) [8].

Despite promising results for the sunitinib group, the CARMENA trial was not without fault. The trial used the MSKCC risk stratification (a system comprising of tumor histology, degree of cancer related symptoms, T stage at diagnosis, and tumor size designed to predict likelihood of recurrence in the 5 years following surgical treatment for RCC first published in 2001) [9]. This differs from the more contemporaneous International mRCC Database Consortium (IMDC) risk which utilizes Karnofsky performance status, and time from diagnosis to start of systemic therapy along with hemoglobin, neutrophil, platelet, and corrected calcium levels to risk stratify patients to help determine treatment [10]. Since its publication, critics have also called into question the trial’s early closure due to poor accrual leading to underpowering. Additionally, crossover between the two treatment arms has been noted. Moreover, CARMENA focused on MSKCC intermediate and poor risk patients, leaving the question of efficacy of CN in favorable risk patients unanswered. A re-analysis of CARMENA by the authors indicated that those with 1 IMDC risk factor had a longer overall survival with CN + sunitinib (31.4 months) vs. sunitinib alone (25.2 months; HR 1.30, p=0.2) [11].

Published in 2019, shortly after CARMENA, was SURTIME which assessed optimal timing of CN combined with TKI therapy. This phase 3 trial set out to investigate upfront CN followed by a course of sunitinib vs sunitinib followed by CN. Like CARMENA, the trial struggled with accruing participants and eventually closed prematurely. To this end, the primary endpoint was altered from PFS to intention to treat 28-week progression free rate (PFR). SURTIME found a 28-week PFR of 42% in the upfront CN arm vs 43% in the deferred CN group (n=49, p=0.61) revealing no improvement in 28-week PFR in the deferred CN group. The study did, however, discover median OS of 32.4 months in the deferred CN arm (95% CI, 14.5–65.3 months) vs 15.0 months in the immediate CN arm (95% CI, 9.3–29.5 months; OS HR 0.57, 95% CI, 0.34–0.95, p=0.03) [12]. Interestingly, the results of both trials proved contradictory to those of several retrospective studies published around the same time which revealed benefit of upfront CN in the TKI era with upfront CN exhibiting better OS [13,14].

Importantly, as the results of CARMENA, SURTIME and the population-based studies above became available, first line treatment for mRCC continued to evolve, moving away from use of TKIs towards utilization of a new class of drugs, immune checkpoint inhibitors (ICIs) [15].

No RCTs have yet been reported in the ICI era. Nonetheless, data from IMDC assert that upfront CN may retain relevance in the treatment paradigm even in the age of ICI. This work, which assessed over 4,000 patients from the IMDC showed that upfront CN was associated with improved OS in both the 437 patients receiving ICI (HR 0.61; 95% CI, 0.41–0.90, p = 0.013) and the 4,202 patients receiving targeted therapy (HR 0.72; 95% CI, 0.67–0.78, p < 0.001) with no differences in OS seen between the two systemic therapies [16].

Additionally, in spite of a lack of prospective data, current American Society of Clinical Oncology guidelines support CN as a viable treatment option in select patients with mRCC asserting that “Cytoreductive nephrectomy may be offered to select patients with kidney-in-place and favorable- or intermediate-risk disease” These guidelines go on to state that optimal candidates for CN include those with the majority of their tumor burden confined to the kidney, good performance status, and no metastases to the brain, bone, or liver. They also specify that CN is best performed by high volume surgeons as part of disease management with an experienced multi-disciplinary team [17].

## Ongoing Clinical Trials

Many of the trials mentioned above that support continued use of CN in current treatment are retrospective and therefore must be considered with caution. These trials are notably limited due to their observational nature which inherently predisposes to confounders such as selection bias. This further highlights the need for additional prospective studies on this topic to fill in gaps in knowledge and provide more reliable and standardized treatment for patients.

Fortunately, as treatment for patients with mRCC continues to advance, prospective trials are enrolling which delve further into the nuance of CN as a part of the multimodal treatment landscape. One such trial, PROBE (NCT04510597), seeks to provide level I evidence regarding benefit of CN vs systemic therapy alone with current standard of care treatment with ICI/ICI or ICI/TKI therapy. Patients enrolled in this trial will receive 10–14 weeks of systemic therapy initially. Those who are found to have progressive or stable disease at this time and are judged to be an appropriate candidate for CN by a qualified urologist will be randomized to continue systemic therapy alone or receive CN followed by additional therapy [18]. Similarly, NORDIC-SUN (NCT03977571) plans to randomize patients pre-treated with ipilimumab/nivolumab with resectable disease thereafter to CN [19].

Cyto-KIK, a trial which began enrolling in 2021, (NCT04322955), assesses the efficacy of cabozantinib and nivolumab prior to and following CN performed at 12 weeks with the primary endpoint being rate of complete response [20].

Finally, SAMURAI (NCT05327686), examines stereotactic ablative radiation therapy in lieu of CN in patients with unresected mRCC receiving ICI therapy who are unable or unwilling to undergo CN [21].

## Indications and Perioperative Considerations

Regardless of timing or choice of systemic therapy, the decision as to which patients ultimately receive CN is complex. Important considerations include disease burden, patient response to systemic therapy, performance status, surgical candidacy, and presence of life altering symptoms such as intractable pain or hematuria [22,23].

Surgery after TKI and/or IO therapy will likely continue to increase whether in locally advanced or mRCC populations. Certain perioperative concerns need to be addressed prior to surgery. One important consideration is the timing of surgery after cessation of systemic therapy. TKIs, due to their anti-angiogenic properties, have been associated with poor wound healing [24–26]. Early studies revealed that surgeries after TKI therapy had higher 30 and 90 day complication rates [27,28]. Consequently, these drugs are frequently held during the perioperative period for a timeframe typically dictated by the drug half-life. ICIs are not governed by the same restrictions and do not typically require a standard washout time prior to surgical intervention though delay to surgery may be needed for patients experiencing common immune related adverse events, particularly those that require high dose steroid treatment [29] (Table 1).

Although ICIs do not harbor the same perioperative complication profile as TKIs, they possess their own side effects and perioperative considerations. The principal concerns in patients receiving presurgical ICIs involves considerations indicated in (Table 1) including desmoplastic reaction which can make CN more challenging. The extent of desmoplastic reaction that will be encountered during nephrectomy is difficult to assess preoperatively. Locally advanced renal cell carcinoma naturally creates a baseline desmoplastic reaction; thus, surgeons don’t know how much preoperative systemic therapy independently contributes to the reaction encountered in a particular case. More work is needed to understand whether choice of systemic therapy, duration of treatment, degree of response, or other factors have implications for the desmoplastic reaction encountered during nephrectomy. Fortunately, most data indicate that clinical outcomes are unaffected by pre-surgical ICI.

One phase I trial examining patients who underwent CN following three doses of nivolumab documented no intraoperative tissue changes and no Clavien 3 or greater post-operative complications. Another retrospective analysis of 113 patients from five US academic centers with locally advanced or mRCC who underwent nephrectomy following ICI treatment showed that intraoperative complication rate, EBL, and operative time were unchanged by exposure to ICI [39]. Several case studies evaluating CN in patients who have undergone treatment with ICI and/or TKI therapy mirror these findings [40,41].

The reasons that pre-surgical ICIs do not appear to significantly impact perioperative outcomes are multifaceted. The desmoplastic reaction is often relatively limited, the renal surgeon performing cytoreductive surgery is generally used to dealing with significant desmoplastic reactions, the benefit of downstaging following preoperative systemic therapy may counter any detrimental effects of a desmoplastic reaction, and most patients that undergo preoperative systemic therapy do not sustain adverse effects that impact perioperative outcomes.

In an analysis of 752 patients receiving cytoreductive nephrectomy from the National Surgical Quality Improvement Program, there were no significant differences in any perioperative outcomes between patients receiving preoperative systemic therapy (n=166) compared to those who underwent upfront nephrectomy (n=586) [42]. Relevant perioperative outcomes are detailed in (Table 2). Patients receiving preoperative systemic therapy were more likely to be on preoperative steroids (23% vs 7%). This may relate to immune checkpoint inhibition (ICI) toxicities and has implications for perioperative management.

Prospective data from the Cyto-Kik study mentioned above also demonstrate the safety of pre-surgical nivolumab and cabozantinib [20]. In this phase 2 trial, participants were treated with 12 weeks of cabozantinib (40 mg daily) and nivolumab (480 mg q4 weeks) prior to undergoing CN. In a recent report of 14 patients that had undergone nephrectomy in this study, no treatment-related surgical complications were noted and there were no delays in resuming systemic therapy after surgery [20].

Ghoreifi et al. have comprehensively reviewed the literature detailing 7 additional series reporting single- and multi-institutional outcomes for undergoing nephrectomy following ICI therapy (n=215 nephrectomies) [23]. Intraoperative complications were noted in 2–19% of cases, 90-day postoperative complications were noted in 14–36%, and mortality rate was 0–9%.

More recently, Reese *et al*. reported data from Memorial Sloan Kettering Cancer Center on a series of 220 patients who underwent cytoreductive nephrectomy between 2015 and 2022, 46 (21%) of whom received ICIs preoperatively. There were no differences in 90-day surgical complications between groups (OR 1.82, 95% CI 0.59–5.14, p=0.3). Interestingly, there was an association between upfront immunotherapy and odds of requiring blood transfusion (OR 4.53, 95% CI 1.82–11.7; p=0.001) but causality cannot be assessed in this observational study [43].

RCC with inferior vena cava tumor thrombus (IVC-TT) may be a particularly appealing niche for pre-surgical ICI. IVC-TT ranges from tumor that protrudes minimally into the IVC (level 1) to bulky tumors that extend to the right atrium (level 4). Unsurprisingly, surgical complication rates relate to the extent of inferior vena cava involvement of IVC-TT [44]. Pre-surgical therapy can downstage IVC-TT [45] which can have significant implications for operative approach and perioperative outcomes [46–48]. Despite this, upfront surgery is still usually performed in both localized and metastatic IVC-TT [49] because of low response rates of TKIs alone. With the higher response rates seen with doublet therapy, we are enthusiastic pre-surgical ICI may eventually have a role for complex IVC-TT. In the authors’ experience, significant IVC-TT complications or progression during doublet ICI therapy is rare. Additionally, downstaging IVC-TT can make surgery less invasive by reducing the extent of vascular clamping required as well as increasing the proportion of cases amenable to robotic IVC thrombectomy. Feasibility of pre-surgical ICI for IVC-TT has been described [50]. A number of ongoing prospective studies mentioned above include participants with IVC-TT including NCT05319015 [51], Cyto-Kik [20], NORDIC-SUN [19] and PROBE [18].

## Quality of Life Considerations

Yet another factor to consider for this patient population is quality of life (QOL) concerns. While life altering symptoms represent an indication to pursue treatment including CN as well as systemic therapy, these treatments are not without drawbacks. While many of these considerations for TKIs and ICIs as well as possibility of surgical complications are mentioned above, all these elements as well as psychological stress of treatment and patient financial burden are all important considerations when determining the best treatment for individuals.

Recent ASCO guidelines for management of metastatic ccRCC highlight that in light of “daunting median survival odds” providers are encouraged to assess patient goals of care early on and consider including palliative care even for patients pursuing active treatment [17].

These guidelines also focus on the financial toxicity that can be common in this patient population stating that mRCC patients undergoing systemic treatment face higher deductibles and increased cancer related costs over time which may decrease patient adherence to treatment. The authors also astutely mention that these patients not only face the financial burdens of direct costs of treatment, but also indirect costs such as missed work and travel to and from appointments. In these instances, shared decision making and collaboration is of paramount importance [17].

## Conclusion

CN following immunotherapy is safe and beneficial in appropriately selected patients. The choice to perform CN in patients with mRCC amidst an ever-changing treatment landscape is nuanced. Clinical trial enrollment is critical to refine selection criteria and timing of CN. As treatment options continue to progress, shared decision-making and multidisciplinary collaboration remain paramount in selecting the optimal treatment course for each patient.

## Figures and Tables

**Table 1.** Systemic therapy for mRCC based on risk category, mechanism, half-life, adverse events.

Treatment	Mechanism of Action	Drug Half Life	Perioperative Hold Time	Adverse Events	Clinical Trials
Axitinib + Pembrolizumab	Axitinib: Inhibits tyrosine kinase receptors VEGFR-1, −2, and −3; decreases angiogenesis, tumor growth, and metastases [30]	Axitinib: 2.5 – 6.1 hrs	Axitinib: minimum 24 hrs. Recommended hold time is ~5 half lives for TKI (12.5–30.5 hrs) [18]	Axitinib (TKI): Anemia, INR increase, thrombocytopenia, lymphocytopenia, thrombo-embolic events, macropapular rash, impaired wound healing [16,30]	KEYNOTE-426 (NCT02853331): Axitinib + Pembrolizumab showed improved PFS, OS, and objective response rate vs. Sunitinib in patients with advanced RCC and no prior treatment [32]
	Pembrolizumab: Monoclonal antibody; binds PD-1 receptor blocking interaction with PD-L1 and -L2; decreases PD-1 mediated immune inhibition [31]	Pembrolizumab: 22 d	Pembrolizumab: No need to hold ICI perioperatively unless due to ongoing AE [16]	Pembrolizumab (ICI): Immune mediated events including colitis, meningitis, pneumonitis, dermatitis, hepatitis, etc. which may require corticosteroid treatment, desmoplastic reaction [31]	
Cabozantinib + Nivolumab	Cabozantinib: Inhibits MET, AXL, and VEGFR decreasing angiogenesis, invasiveness, metastasis, and immunomodulation of tumor microenvironment [33]	Cabozantinib: 55–99 hrs	Cabozantinib: Recommended hold time is ~5 half lives for TKI (11.5–20.6d)	Cabozantinib (TKI): See TKI mediated adverse events listed under Axitinib above	CheckMate 9ER (NCT03141177): Cabozantinib + Nivolumab showed improved PFS, OS, and objective response vs. Sunitinib in patients with advanced RCC with no prior treatment [34]
	Nivolumab: Monoclonal antibody; binds PD-1 receptor blocking interaction with PD-L1; decreases PD-1 mediated immune inhibition [34]	Nivolumab: ~25 d	Nivolumab: No need to hold ICI perioperatively unless due to ongoing AE [16]	Nivolumab (ICI): See ICI mediated adverse events listed under Pembrolizumab above	
Lenvatinib + Pembrolizumab	Lenvatinib: Inhibits tyrosine kinase receptors VEGFR 1–3, FGFR 1–4, KIT, RET, and PDGRFα, decreases angiogenesis, lymphogenesis, tumor growth, and metastases [35]	Lenvatinib: ~28 hrs	Lenvatinib: Recommended hold time is ~5 half lives for TKI (5.8d)	Lenvatinib (TKI): See TKI mediated adverse events listed under Axitinib above	CLEAR Trial (NCT02811861): Lenvatinib + Pembrolizumab showed improved OS and PFS vs. Sunitinib in patients with advanced RCC [36]
	Pembrolizumab: Monoclonal antibody; binds PD-1 receptor blocking interaction with PD-L1 and -L2; decreases PD-1 mediated immune inhibition [29]	Pembrolizumab: 22 d	Pembrolizumab: No need to hold ICI perioperatively unless due to ongoing AE [16]	Pembrolizumab (ICI): See ICI mediated adverse events listed under Pembrolizumab above	
Ipilimumab + Nivolumab	Ipilimumab: Monoclonal antibody; binds to CTLA-4 and blocks interactions with CD80/CD86; helps activate cytotoxic T cells; reduces T-regulatory cell function [37]	Ipilimumab: 15.4 d	Ipilimumab and Nivolumab: No need to hold ICI perioperatively unless due to ongoing AE [16]	Ipilimumab (ICI): See ICI mediated adverse events listed under Pembrolizumab above	CheckMate 214 (NCT02231749): Nivolumab + Ipilimumab showed improved OS and objective response vs. Sunitinib in intermediate and poor risk patients with mRCC and no prior treatment [38]
	Nivolumab: Monoclonal antibody; binds PD-1 receptor blocking interaction with PD-L1; decreases PD-1 mediated immune inhibition [34]	Nivolumab: ~25 d		Nivolumab (ICI): See ICI mediated adverse events listed under Pembrolizumab above	

**Table 2.** Perioperative outcomes of patients receiving cytoreductive nephrectomy in the National Surgical Quality Improvement Program database in the era of immune checkpoint inhibitor use in renal cell carcinoma (2019–2021).

Outcome	Upfront CN	Deferred CN	P
	N=586	N=166	
Major complications	8%	5%	0.188
Overall complications	33%	39%	0.152
Infectious complications	7%	5%	0.518
Median total operative time (mins)	172	184	0.14
MIS →Open conversion	6%	4%	0.517
Adjunctive procedures	31%	34%	0.434
Discharge to care facility	5%	2%	0.152
Length of total hospital stay (days)	3	3	0.914
